# Targeting ATF4-dependent pro-survival autophagy to synergize glutaminolysis inhibition

**DOI:** 10.7150/thno.60028

**Published:** 2021-07-25

**Authors:** Shuting Han, Liyuan Zhu, Yiran Zhu, Yuan Meng, Jiaqiu Li, Ping Song, Neelum Aziz Yousafzai, Lifeng Feng, Miaoqin Chen, Yanmei Wang, Hongchuan Jin, Xian Wang

**Affiliations:** 1 Department of Medical Oncology, Sir Run Run Shaw Hospital, Medical School of Zhejiang University, Hangzhou, China; 2 Laboratory of Cancer Biology, Key Lab of Biotherapy, Cancer Center of Zhejiang University, Sir Run Run Shaw Hospital, Medical School of Zhejiang University, Hangzhou, China; 3 Department of Allied Health Sciences, University of Poonch Rawalakot, AJK, Pakistan

**Keywords:** Glutaminolysis inhibition, ATF4, DDIT4, N^6^-methyladenosine, metabolic synthetic lethality

## Abstract

As glutamine plays a central role in cancer metabolism, inhibition of glutaminolysis has become an ideal anticancer therapeutic target. However, glutaminolysis inhibition leads to activation of autophagy, which compromises its antitumor effect. Hence, we investigated the mechanism underlying glutaminolysis inhibition-induced pro-survival autophagy.

**Methods:** High-throughput sequencing was performed on colorectal cancer (CRC) cells before and after glutaminolysis inhibition to identify differentially expressed genes. Activating transcription factor 4 (ATF4) pathway enrichment in glutaminolysis inhibited cells was identified through gene set enrichment analysis. ATF4 expression was assessed by quantitative real-time PCR (qRT-PCR) and western blotting. The function of ATF4 on mechanistic target of rapamycin (mTOR) regulation was assessed by western blotting. Luciferase reporter assays and chromatin immunoprecipitation were used to confirm the regulation of DNA damage inducible transcript 4 (DDIT4) by ATF4. mRNA half-life assays, RNA immunoprecipitation, qRT-PCR and western blotting were performed to determine the relationship between FTO alpha-ketoglutarate dependent dioxygenase (FTO), YTH N^6^-methyladenosine RNA binding protein 2 (YTHDF2), and ATF4. ATF4 regulation of pro-survival autophagy was measured by tandem monomeric red fluorescent protein-green fluorescent protein fluorescence microscopy. Finally, the synergistic effect of autophagy and glutaminolysis inhibition was analyzed in an azoxymethane/dextran sodium sulfate mouse model.

**Results:** The ATF4 pathway was activated in CRC cells upon glutaminolysis inhibition. Functionally, ATF4 transcriptionally upregulated DDIT4 to suppress mTOR, which induced pro-survival autophagy during glutaminolysis inhibition. Interestingly, glutaminolysis inhibition promoted *ATF4* mRNA expression by abrogating N^6^-methyladenosine (m^6^A) modification and YTHDF2-mediated RNA decay. Finally, inhibition of ATF4-induced autophagy enhanced the antitumor efficacy of glutaminolysis inhibition.

**Conclusion:** Glutaminolysis inhibition upregulated *ATF4* expression in an m^6^A-dependent manner to activate pro-survival autophagy through transcriptional activation of the mTOR inhibitor DDIT4. Targeting ATF4-induced autophagy is a new strategy to synergize glutaminolysis-targeting therapies for cancer treatment.

## Introduction

Colorectal cancer (CRC) is the third most prevalent cancer and has the second highest mortality worldwide 1. Apart from the traditional surgery and chemotherapy, several new drugs targeting vascular endothelial growth factor (VEGF) 2 and epidermal growth factor receptor (EGFR) 3 have been used in combination with chemotherapy to treat patients with advanced CRC. However, such treatments are not satisfactory in most cases; therefore, more efficient therapies need to be developed from a better understanding of colorectal carcinogenesis 4.

Due to the demands of cell growth and proliferation, cancer cells require an excessive amount of nutrients. Often, cellular metabolism is reprogrammed to confer selective advantages. In addition to the well-known Warburg effect, enhanced glutaminolysis in cancer cells is attracting more attention since glutamine is the most abundant amino acid in circulation 5. As a donor of both carbon and nitrogen, glutaminolysis facilitates energy generation and biomass accumulation 6. Upregulated glutaminolysis has been discovered in various cancer types 7. In proliferating cancer cells, glutamine is preferentially consumed about 10 times more than other amino acids 8. Glutamine addiction in cancer cells makes glutamine metabolism an excellent therapeutic target. The rate-limiting step in glutaminolysis is the conversion of glutamine to glutamate, which is catalyzed by glutaminase (GLS). Elevated expression of GLS has been found in various cancers 9. We recently reported that GLS1 is epigenetically upregulated in colorectal carcinogenesis 10, 11. Therefore, the high reliance on glutamine of cancer cells makes targeting GLS to inhibit glutaminolysis a promising therapy option for human cancers including CRC. However, inhibition of glutaminolysis could initiate pro-survival autophagy via mechanistic target of rapamycin (mTOR) inactivation 10. mTOR is a serine/threonine kinase that plays a central role in cell growth and proliferation control 12. mTOR activity is tightly regulated through several pathways including the growth factor pathway, AMP-activated protein kinase (AMPK) pathway, DNA integrity, and hypoxia 13. In recent years, glutamine has been recognized to be essential for mTOR activation 14. Glutamine can drive mTORC1 translocation and activation at the lysosome membrane independently of RagA and RagB 15. Nevertheless, the intracellular concentration of glutamine may increase as a consequence of glutaminolysis inhibition, which would activate mTOR. Therefore, we suspect the presence of additional pathways that actively inhibit mTOR activation.

In this study, we demonstrate that activating transcription factor 4 (ATF4) is upregulated during glutaminolysis inhibition to inactivate mTOR signaling through transcriptional activation of the mTOR suppressor DNA damage-inducible transcript 4 (DDIT4). In response to glutaminolysis inhibition, the N^6^-methyladenosine (m^6^A) modification eraser FTO alpha-ketoglutarate dependent dioxygenase (FTO) is upregulated to reduce m^6^A modification of *ATF4* mRNA, thus compromising YTH N^6^-methyladenosine RNA binding protein 2 (YTHDF2)-dependent mRNA degradation to increase the expression of* ATF4* mRNA. As a result, targeting pro-survival autophagy is synthetically lethal with glutaminolysis inhibition in the treatment of CRC in vivo.

## Materials and Methods

### Cell culture

The CRC cell lines SW480 and HCT116 were purchased from Type Culture Collection of the Chinese Academy of Science (Shanghai, China). SW480 cells were cultured in RPMI 1640 Medium (118575-093, Invitrogen, Shanghai, China) supplemented with 10% fetal bovine serum (10099141C, Gibco, Shanghai, China) and 100 μg/mL antibiotics (100 U/mL penicillin and 100 μg/mL streptomycin, Gibco). HCT116 cells were cultured in McCoy's 5A Medium (16600-082, Gibco). During glutamine starvation, SW480 cells were cultured in glutamine-free RPMI 1640 Medium (42401018, Gibco). HCT116 cells were cultured in glutamine-free McCoy's 5A Medium (PM150713, Procell, Wuhan, China). The cells were grown in a humidified incubator with 5% CO_2_ and 95% humidity at 37 °C.

### Western blotting

Cell lysates were collected with loading buffer containing 2% sodium dodecyl sulfate, 10% glycerinum, 0.1% bromophenol blue, 1.5% dithiothreitol, and 0.1 M Tris-HCI (pH 6.8). The cell lysates were boiled loaded on SDS-PAGE gels transferred to polyvinylidene fluoride membranes (Bio-Rad Laboratories, Shanghai, China), and blocked with 5% non-fat milk in Tris-buffered saline with 0.1% Tween^®^ 20 detergent (TBST). The blots were incubated with the indicated primary antibodies at 4 °C overnight. The next day, the blots were washed with TBST and incubated with the suitable secondary antibody (111-035-003, Jackson Immuno Research, West Grove, PA, USA) at room temperature for 2 h. The blots were then visualized on a Bio-Rad ChemiDOC Touch Imaging System (Bio-Rad Laboratories, China) with a Chemiluminescence Detection Kit for HRP (20-500-120, Biological Industries, Cromwell, CT, USA; FD8030, FDbio Science, China) for detection.

### RNA extraction and quantitative real-time PCR (qRT-PCR)

Total RNA was isolated using TRIzol^TM^ Reagent (15596026, Invitrogen) and quantified with a NanoDrop 2000^TM^ (Thermo Fisher Scientific, Wilmington, DE, USA). 2 μg RNA was used for the reverse transcription reaction with High-Capacity cDNA Reverse Transcription Kit (Thermo Fisher Scientific Inc., Shanghai, China) according to the manufacturer's protocols. qRT-PCR was performed using SYBR^TM^ Green Master Mix Kit and a LightCycler^®^ 480 II system (Roche, Shanghai, China) for mRNA expression determination. β-Actin was used for the normalization. The primers used in the study are listed in Table S1.

### Antibodies and chemicals

ATF4 (rabbit, 11815S), p-mTOR (rabbit, 5536S), mTOR (rabbit, 2983S), p-p70S6K (rabbit, 9205S), p70S6K (rabbit, 2708S), pS6 (rabbit, 4857S), LC3B (rabbit, 3868S), and sequestosome 1 (SQSTM1, rabbit, 8025) monoclonal antibodies were purchased from Cell Signaling Technology (Shanghai, China). DDIT4 (rabbit, 10638-1-AP) and YTHDF2 (rabbit, 24744-1-AP) polyclonal antibodies were purchased from Proteintech. FTO (rabbit, ab126605), WTAP (rabbit, ab195380), ALKBH5 (rabbit, ab69325), and m^6^A (mouse, ab208577) antibodies were purchased from Abcam. METTL3 (rabbit, a8370), and METTL14 (rabbit, a8530) polyclonal antibodies were purchased from ABclonal. Compound 968 was purchased from Sigma-Aldrich (SML1327). Actinomycin D was purchased from Selleck Chemicals (S8964). Chloroquine (CQ) was obtained from Sigma-Aldrich (C6628). Meclofenamate sodium was purchased from MCE (HY-B1320). CB839 was purchased from Selleck Chemicals (S7655).

### siRNA and plasmid transfection

For siRNA transfection, cells were seeded in six-well plates overnight and transfected with Lipofectamine^TM^ RNAiMAX transfection reagent (133778, Thermo Fisher Scientific) according to the manufacturer's protocol. For plasmid transfection, cells were seeded overnight and the plasmids were transfected with X-tremeGENE^TM^ HP DNA Transfection Reagent (06366236, Merck/Sigma Aldrich) according to the manufacturer's protocol. Full-length ATF4 plasmid and DDIT4 plasmids were purchased from Gene Pharma Company (Shanghai, China). Mutant ATF4 was cloned into an EXO5-flag vector. All plasmids were purified using EndoFree Plasmid Maxi Kit (QIAGEN). siRNAs were designed and synthesized by Gene Pharma Company (Shanghai, China). All siRNA sequences used for knockdown are listed in Table S2.

### Cell counting assay

Cells were plated overnight in 12-well plates then treated with compound 968 (25 μM) for 72 h. The cells were stained with 0.4% trypan blue and counted with an automated cell counter (Bio-Rad Laboratories).

### Apoptosis detection

Cell apoptosis was detected by flow cytometer. Cells were treated with compound 968 (25 μM) for 72 h, harvested with trypsin, and re-suspended in 100 μL of of 1× binding buffer. 5 μL of FITC annexin V and propidium iodide staining solution (556547, BD Biosciences, USA) was added to the cell suspension and incubated for 15 min at room temperature. After dilution with 400 μL of 1× binding buffer, the samples were analyzed using a FACSCalibur^TM^ flow cytometer (BD Biosciences).

### Luciferase assay

Cells transiently expressing* Renilla* luciferase and *firefly* luciferase reporter plasmids were treated with compound 968. The luciferase activity of the cell lysates was determined luminometrically using a GloMax^®^ 20/20 Luminometer (E5311, Promega) with a dual luciferase assay system (Promega) according to the manufacturer's protocol.

### Metabolite analysis

Cells were seeded in 6-well plates overnight and treated with compound 968 (25 μM). The concentration of medium glutamine was measured using Glutamine Assay Kit (KA1670, Abnova), and α-KG was measured using an assay kit (KA0872, Abnova). In vivo glutamate was measured using Glutamine/Glutamate-Glo^TM^ Assay (J8021, Promega). In vivo glutathione (GSH) was measured using a colorimetric assay kit (ab239709, Abcam). All assays were performed according to the manufacturer's protocol.

### Tandem monomeric red fluorescent protein-green fluorescent protein (mRFP-GFP) fluorescence microscopy

For tandem fluorescent LC3 puncta detection, cells were transfected with tfLC3 plasmid and ATF4 siRNA for 48 h. Then the transfected cells were reseeded on glass coverslips in 12-well plates overnight. The next day, the cells were treated with dimethyl sulfoxide (DMSO) or compound 968 (25 μM) for 24 h. The cells were sealed with 20% glycerinum and visualized using a Nikon A1 laser scanning confocal microscope. LC3 dots per cell were counted using ImageJ.

### Chromatin immunoprecipitation (ChIP)

Cells seeded overnight in 10 cm dishes were treated with DMSO or compound 968 (25 μM). ChIP analysis was performed using SimpleChIP^TM^ Enzymatic Chromatin IP Kit (9180s, Cell Signaling Technology) as previously described 16. The antibodies used were anti-ATF4 or control rabbit IgG. The primers used for qRT-PCR of precipitated DNA are listed in Table S1.

### Clone formation assay

800 cells were seeded in 6-well plates overnight and then treated with DMSO or compound 968 (25 μM) for 12 days. The medium was then removed and the cells were washed with PBS. For fixation 4% paraformaldehyde was applied for 20 min at room temperature. The clones were then stained with 0.2% crystal violet solution for visualization.

### RNA half-life assay

Cells were first treated with siRNA for 48 h and then treated with actinomycin D (5 μg/mL) to block new RNA synthesis. Cells were then harvested for total RNA extraction at indicated time points. The samples were analyzed by qRT-PCR.

### RNA immunoprecipitation (RIP) assay

RIP was performed using Magna RIP^TM^ RNA-Binding Protein Immunoprecipitation Kit (17-700, Millipore). Cells were seeded in 10cm dishes, treated with the indicated treatments, then lysed in 100 μL RIP lysis buffer with added RNase and protease inhibitors. The antibodies of interest and protein G magnetic beads were incubated at room temperature and washed with washing buffer. Cell lysate was added to the antibody-linked beads and incubated overnight at 4 °C. The samples were then washed six times with washing buffer and protein was digested at 55 °C. Total RNA was isolated from the aqueous solution after digestion and quantified by qRT-PCR.

### Animal experiments

Animal care and experiments were performed according to the Institutional Animal Care and Use Committee and NIH guidelines. Forty-five 5-week-old male C57BL/6J mice were purchased from Model Animal Research Center of Nanjing University and divided randomly into 9 groups. As the control group, group 1 mice were given sterile saline. In the first week, the other 8 groups were given a signal dose of azoxymethane (AOM) (10 mg/kg, intraperitoneally). From the second week on, the mice were given 2% dextran sodium sulfate (DSS) (diluted in water) for three cycles (one cycle includes one week of 2% DSS and two weeks of DSS-free water). This DSS feeding cycle was carried out 2 times before starting the therapeutic protocol as follows: group 2, DMSO; group 3, CQ; group 4, L-L-asparaginase (L-ASP); group 5, CQ + L-ASP; group 6, compound 968; group 7, compound 968 + CQ; group 8, compound 968 + L-ASP; group 9, compound 968 + CQ + L-ASP (CQ, 50 mg/kg; L-ASP, 1000 IU/kg; compound 968, 10 mg/kg). All injections were performed twice a week for 10 weeks and mice were sacrificed on day 100. The colorectal tissues were dissected, flushed with PBS, and cut open longitudinally along the main axis. The numbers and sizes of tumors were recorded. The tumors were fixed in 4% formalin and prepared for immunohistochemical (IHC) analysis.

### Statistical analysis

All experiments were performed in at least triplicate, and Student's *t*-test or ANCOVA were used for statistical analysis. Differences were regarded as significant when *P* < 0.05.

## Results

### Inhibition of glutaminolysis inactivates mTOR by upregulating ATF4 expression

**Figure 1:**
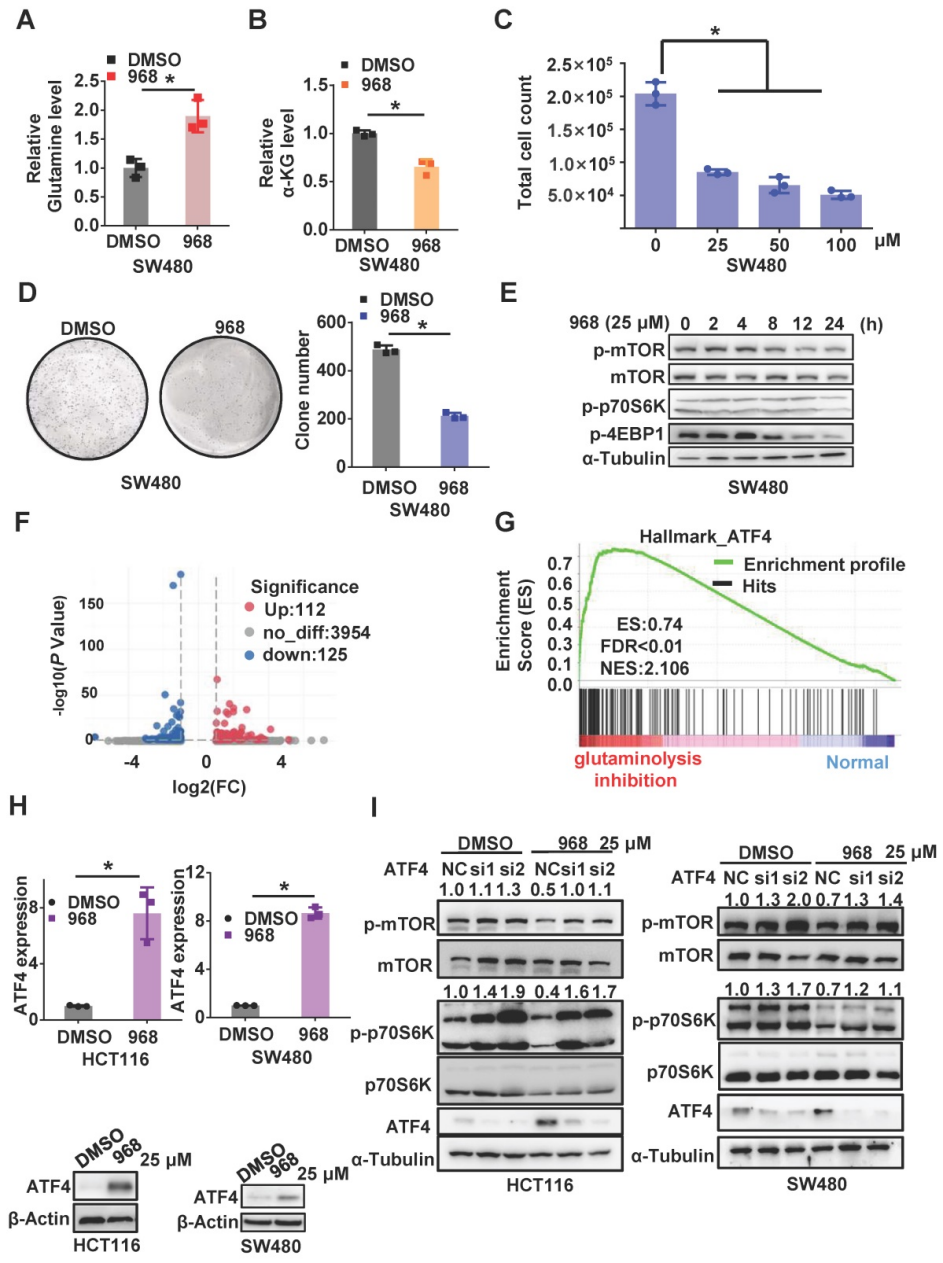
** Inhibition of glutaminolysis inactivates mTOR by upregulating ATF4 expression.** (A-B) The glutamine level in medium (A) and intercellular level of α-KG (B) of cells treated by DMSO or compound 968 (968) (25 μM) for 24 h were detected by metabolic analysis. (C) The effect of 968 on cell viability was determined by cell counting assay. Cells treated with DMSO or 968 (25 μM, 50 μM,100 μM) for 72 h were then counted. Data are shown as the means ± SD from three experiments. (D) The colony formation of SW480 cells treated with 968 at 25 μM. The result presents the number of colonies. (E) The effect of 968 on mTORC1 activity was analyzed by western blotting. (F) Volcano plots of differentially expressed genes in CRC before and after glutaminolysis inhibition. (G) GSEA on differentially expressed genes of normal and glutaminolysis inhibited cells. Red indicated glutaminolysis inhibited cells and blue indicated normal cultured cells. (H) ATF4 expression of cells treated by 968 for 24 h was detected by western blotting and qRT-PCR. (I) The effect of ATF4 knockdown on mTORC1 activity upon 968 treatment was analyzed by western blotting. p-mTOR, p-p70S6K band density was quantified and expressed as fold change, compared with the control, by arbitrarily setting the control value as 1. Data are shown as the means ± SD from three experiments. For all experiments, statistical significance was assessed by Student's *t*-tests, *P < 0.05.

To explore the effect of glutaminolysis inhibition, we first treated CRC cells with compound 968, a small molecule that specifically targets GLS1 17. Glutamine in the cell culture media was elevated in both cell lines after compound 968 treatment, which implies a decline in glutamine usage by the cells (Figure 1A and Figure S1A). Consistently, the concentration of α-KG and glutamate, major downstream metabolites of glutamine, was decreased after compound 968 treatment (Figure 1B and Figure S1B-C). Meanwhile, compound 968 inhibited cell proliferation (Figure 1C and Figure S1G), promoted apoptosis (Figure S1F), and significantly reduced the size and number of colonies (Figure 1D and Figure S1H). These results demonstrated compound 968 treatment is sufficient to inhibit glutaminolysis. Consistent with our previous reports that inhibition of glutaminolysis could activate autophagy, compound 968 downregulated mTOR in a time-dependent manner (Figure 1E and Figure S1I).

To further explore the mechanism by which glutaminolysis inhibition to inactivates the mTORC1 pathway, we performed RNA-sequencing of CRC cells before and after glutaminolysis inhibition (Figure 1F). Gene set enrichment analysis (GSEA) based on differential mRNA expression indicated that the expressions of ATF4 target gene sets were positively correlated with glutaminolysis inhibition (Figure 1G). ATF4 is a stress-induced transcriptional factor that plays a critical role in redox balance and amino acid metabolism maintenance 18. Increased ATF4 expression in CRC cells was observed after glutamine starvation or treatment with the GLS inhibitors compound 968 or CB-839 at both the transcriptional and protein levels (Figure 1H and Figure S2A-D). Interestingly, knockdown of ATF4 in SW480 and HCT116 cells successfully reversed the mTOR inactivation induced by compound 968 (Figure 1I). Taken together, the above results suggest that the stress response protein ATF4 plays an important role in mTOR inactivation after glutaminolysis inhibition.

### Inhibition of glutaminolysis upregulates DDIT4 to inactivate mTOR

**Figure 2:**
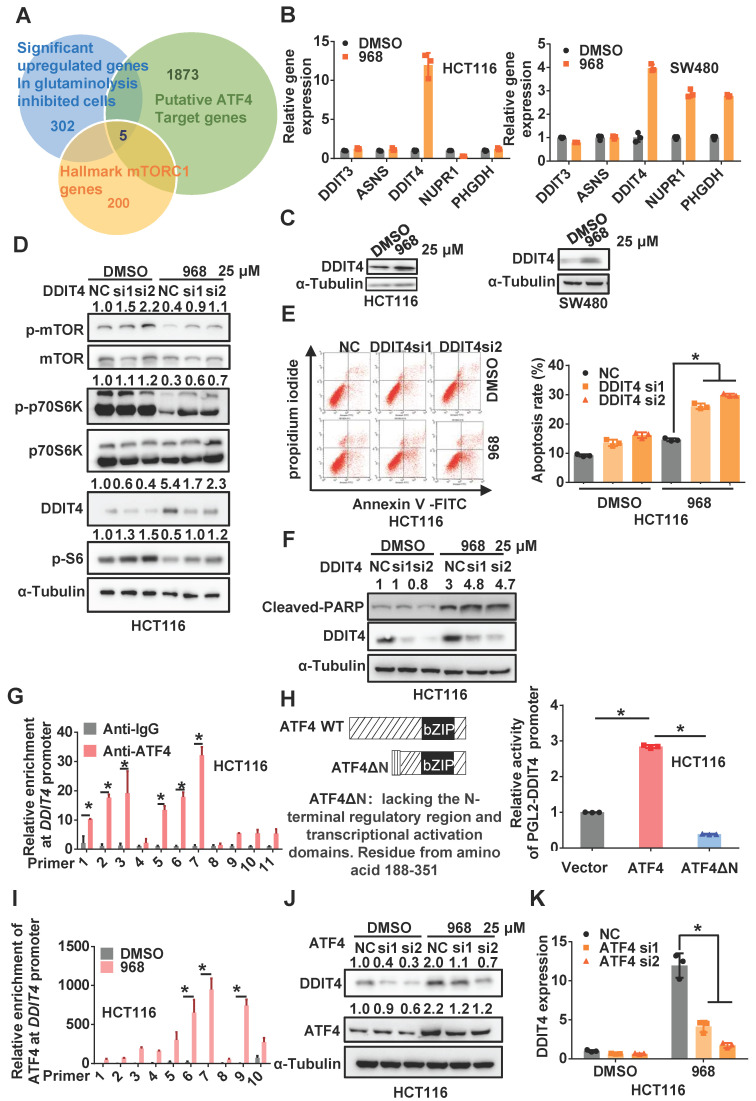
** ATF4 transcriptionally upregulates DDIT4 upon inhibition of glutaminolysis to inactivate mTOR.** (A) Venn diagrams for the intersection of the candidate genes derived from ATF4 potential target genes, differentially expressed genes upon glutaminolysis inhibition, and hallmarks genes of mTORC1. (B) *DDIT3, ASNS, DDIT4, NUPR1, PHGDH* mRNA expression of cells treated by 968 for 24 h was detected by qRT-PCR. (C) DDIT4 expression of cells treated by 968 for 24 h was detected by western blotting. (D) The effect of DDIT4 knockdown on mTORC1 activity of cells treated by 968 for 24 h was detected by western blotting. p-mTOR, p-p70S6K, p-S6, DDIT4 band density was quantified and expressed as fold change, compared with the control, by arbitrarily setting the control value as 1. (E) The effect of DDIT4 knockdown on 968-induced cell apoptosis was measured by Flow Cytometry. The result presents the proportion of apoptotic cells. (F) The expression of cleaved PARP after DDIT4 knockdown and 968 treatment was detected by western blotting, cleaved PARP band density was quantified and expressed as fold change, compared with the control, by arbitrarily setting the control value as 1. (G) ATF4 enrichment at the *DDIT4* promoter was determined with ChIP assay. (H) Schematic representation of ATF4 and dominant-negative ATF4ΔN, an ATF4 mutant lacks the N-terminal transcriptional activation domain was shown. The transcription activation ability of ATF4 and dominant-negative ATF4ΔN on the *DDIT4* promoter was measured with luciferase assay. (I) The change of ATF4 enrichment at the *DDIT4* promoter after 968 treatment was determined with ChIP assay. (J) DDIT4 protein expression after ATF4 knockdown during 968 treatment was detected by western blotting. ATF4, DDIT4 band density was quantified and expressed as fold change, compared with the control, by arbitrarily setting the control value as 1. (K) *DDIT4* mRNA expression after ATF4 knockdown during 968 treatment was detected by qRT-PCR. Data are shown as the means ± SD from three experiments. For all experiments, statistical significance was assessed by Student's* t*-tests, *P< 0.05.

Since ATF4 is a transcriptional factor, we hypothesized that ATF4 might activate target gene transcription to inactivate mTOR signaling. Thus, we used an online tool called ChIP-Atlas 19 to search for potential target genes of ATF4 among genes differentially expressed upon glutaminolysis inhibition and hallmark genes of mTORC1 (Figure 2A). Interestingly, DDIT4 (aka REDD1), which was reported to negatively regulate mTOR activity in a tuberous sclerosis complex 2 (TSC2)-dependent manner 20, was upregulated most in both cell lines among ATF4 target genes (Figure 2B). In addition, DDIT4 expression was elevated in HCT116 and SW480 cells during compound 968 treatment or glutamine starvation (Figure 2C and Figure S3A-B). Moreover, DDIT4 depletion rescued the mTOR inactivation induced by compound 968 treatment (Figure 2D and Figure S3C), while its overexpression indeed inactivated mTOR signaling (Figure S3D). In addition, DDIT4 knockdown increased the apoptosis induced by compound 968, confirming the relevance of DDIT4 upregulation to glutaminolysis inhibition (Figure 2E-F and Figure S3E-F). Taken together, these results suggest that ATF4 upregulates DDIT4 expression to inactivate mTOR signaling in glutaminolysis inhibition.

### ATF4 transcriptionally upregulates DDIT4 upon inhibition of glutaminolysis

According to the online database JASPAR 21, multiple ATF4 binding sites were found on the *DDIT4* promoter (Figure S4A). Indeed, ChIP assays confirmed the physical interaction of ATF4 with the *DDIT4* promoter (Figure 2G and Figure S4B-C). In addition, wild-type ATF4, but not dominant-negative ATF4ΔN lacking the N-terminal transcriptional activation domain, increased the activity of a luciferase reporter driven by the *DDIT4* promoter (Figure 2H and Figure S4D-E), highlighting the dependence of ATF4 on the transcriptional activation of *DDIT4*. Upon glutaminolysis inhibition, the interaction between ATF4 and the *DDIT4* promoter was notably increased (Figure 2I and Figure S4F), which was accompanied by increased enrichment of methylated histone H3 lysine 4 (H3K4) at the *DDIT4* promoter (Figure S4G). Importantly, ATF4 knockdown significantly compromised compound 968-induced DDIT4 upregulation (Figure 2J-K and Figure S4H-I). Therefore, these results indicate that ATF4 transcriptionally upregulates DDIT4 expression in response to glutaminolysis inhibition.

### Inhibition of glutaminolysis stabilizes *ATF4* mRNA by reducing its m^6^A modification

**Figure 3:**
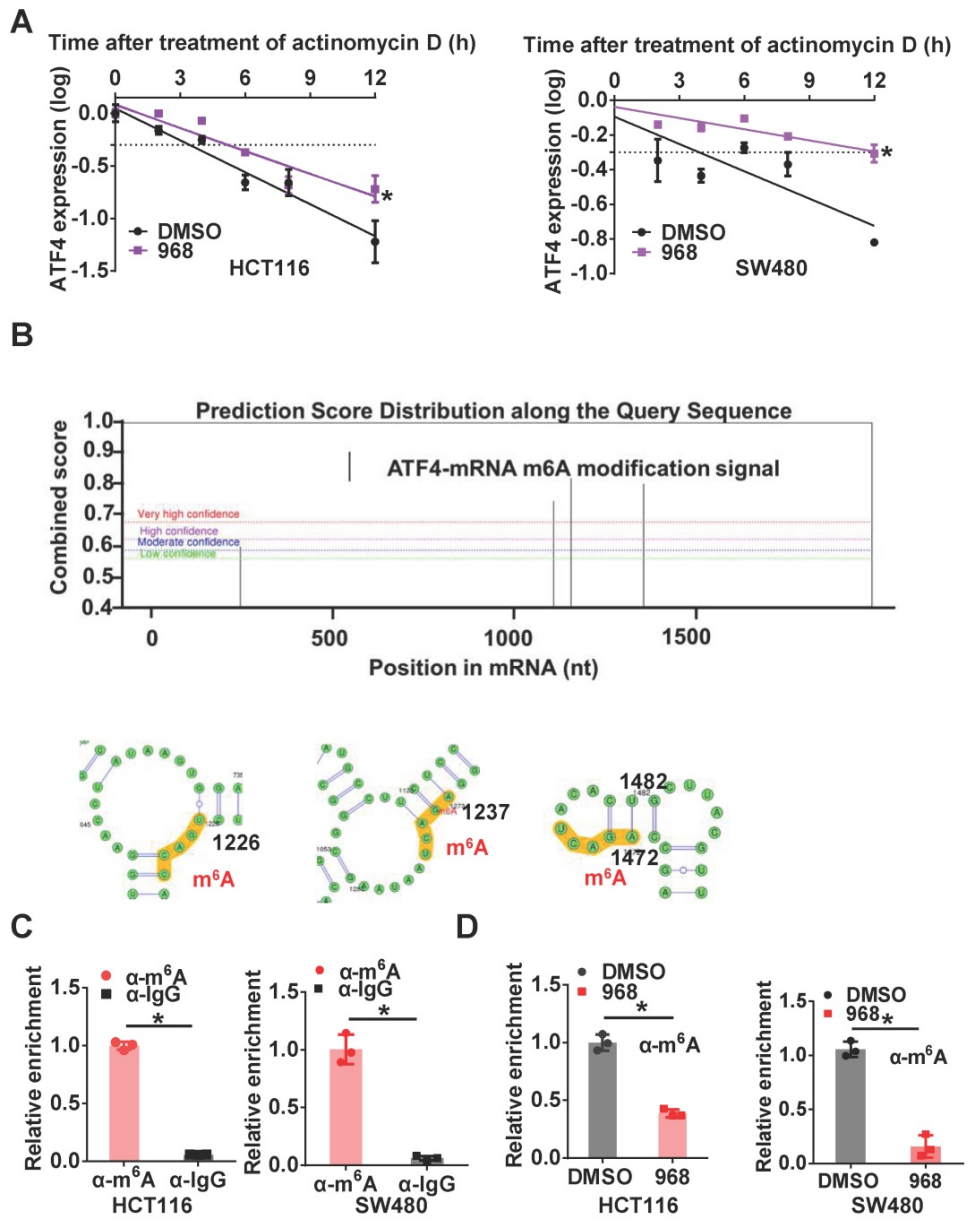
** Inhibition of glutaminolysis stabilizes *ATF4* mRNA by reducing its m^6^A modification.** (A) The change of the half-life of *ATF4* mRNA after 968 treatment was assessed by qRT-PCR. (B) SRAMP was used to screen m^6^A modification sites based on *ATF4* mRNA sequence. Bioinformatic prediction of m^6^A modifications on *ATF4* mRNA secondary structure with SRAMP was shown. (C) The binding capacity of m^6^A to *ATF4* mRNA was assessed by RIP assay. (D) The effect of 968 treatment on m^6^A enrichment at *ATF4* mRNA was assessed by RIP assay. Data are shown as the means ± SD from three experiments. Statistical significance was assessed by ANCOVA analysis in (A), for other experiments, statistical significance was assessed by Student's *t-*tests, *P < 0.05.

To further investigate the mechanism underlying *ATF4* mRNA upregulation after glutaminolysis inhibition, we first compared the stability of *ATF4* mRNA before and after glutaminolysis inhibition. After compound 968 treatment, the half-life of *ATF4* mRNA was extended (Figure 3A). As m^6^A is the most common mRNA modification to regulate also RNA stability 22, we explored the relevance of m^6^A modification to *ATF4* mRNA stability. First, through analysis using the online tool SRAMP 23, several potential m^6^A modification sites were found on *ATF4* mRNA (Figure 3B). Next, m^6^A methylation of *ATF4* mRNA in HCT116 and SW480 cells was confirmed by RIP assays (Figure 3C). Moreover, m^6^A modification of *ATF4* mRNA was decreased after compound 968 treatment (Figure 3D). To sum up, these results demonstrate that m^6^A modification is likely responsible for glutaminolysis-regulated *ATF4* mRNA expression.

### YTHDF2 is responsible for downregulating ATF4 mRNA stability

**Figure 4:**
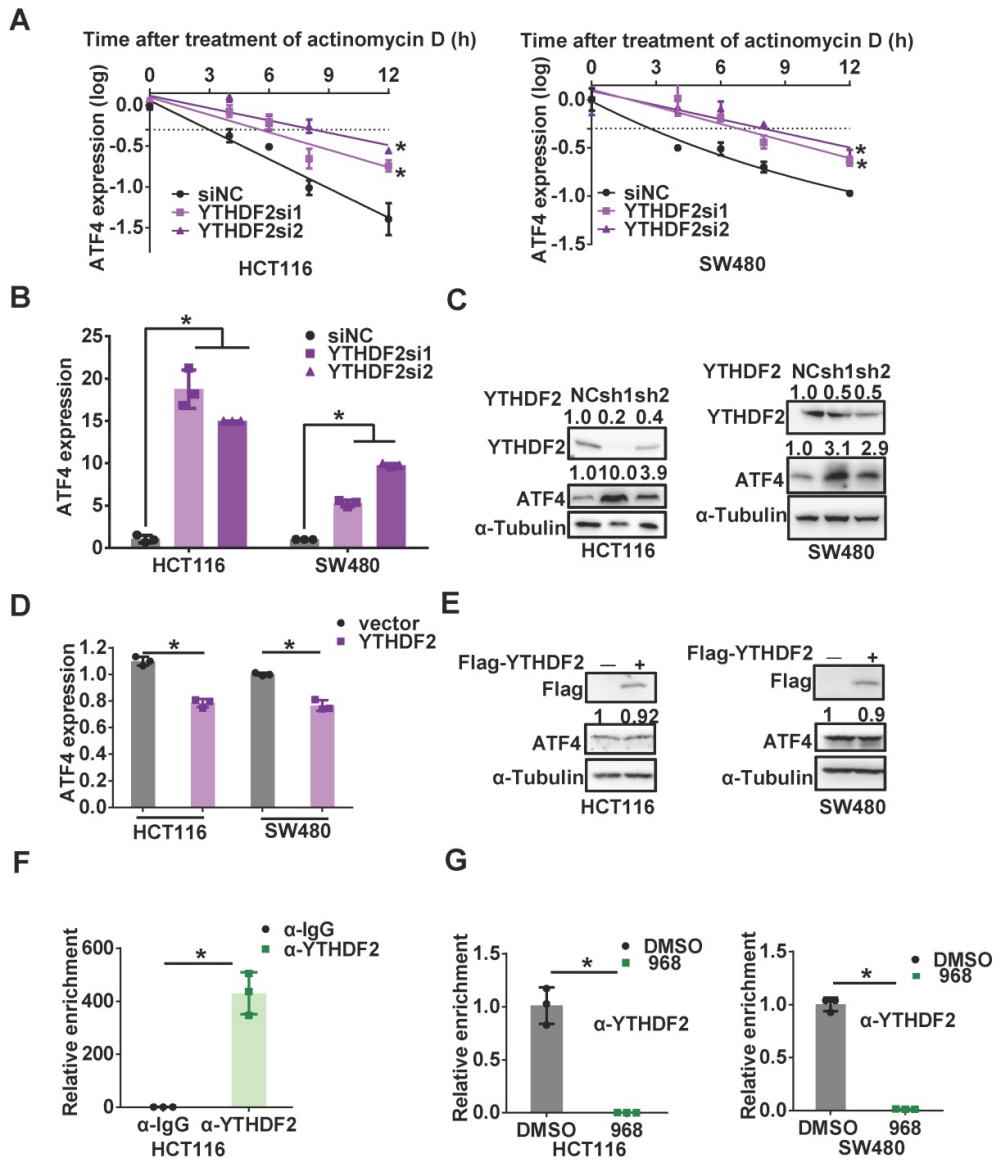
** YTHDF2 is responsible for downregulating *ATF4* mRNA stability.** (A) The change of the half-life of *ATF4* mRNA by YTHDF2 knockdown was assessed by qRT-PCR. (B) *ATF4* mRNA expression with YTHDF2 knockdown was detected by qRT-PCR. (C) ATF4 protein expression with YTHDF2 knockdown was detected by western blotting. YTHDF2, ATF4 band density was quantified and expressed as fold change, compared with the control, by arbitrarily setting the control value as 1. (D)* ATF4* mRNA expression with YTHDF2 overexpression was detected by qRT-PCR. (E) ATF4 protein expression with YTHDF2 overexpression was detected by western blotting. ATF4 band density was quantified and expressed as fold change, compared with the control, by arbitrarily setting the control value as 1. (F) The binding capacity of YTHDF2 to *ATF4* mRNA was assessed by RIP assay. (G) The effect of 968 treatment on YTHDF2 enrichment change on *ATF4* mRNA was assessed by RIP assay. Data are shown as the means ± SD from three experiments. Statistical significance was assessed by ANCOVA analysis in (A), for other experiments, statistical significance was assessed by Student's* t*-tests, *P < 0.05.

The outcomes of m^6^A-methylated RNAs are largely determined by m^6^A readers, among which YTHDF2 has been reported to selectively recognize m^6^A for subsequent degradation of m^6^A-containing mRNA 24. To clarify whether YTHDF2 is responsible for regulating the stability of *ATF4* mRNA, we compared the half-lives of *ATF4* mRNA before and after YTHDF2 knockdown. Knocking down YTHDF2 indeed extended the half-life of *ATF4* mRNA (Figure 4A) and increased *ATF4* mRNA and protein expressions (Figure 4B-C). On the other hand, YTHDF2 over-expression suppressed the ATF4 expression induced by compound 968 (Figure 4D-E). RIP assays further confirmed that YTHDF2 was able to bind *ATF4* mRNA (Figure 4F), and this binding was impaired after compound 968 treatment (Figure 4G). Therefore, the m^6^A reader YTHDF2 is involved in the regulation of *ATF4* mRNA stability in response to glutaminolysis inhibition.

### Inhibition of glutaminolysis upregulates FTO to reduce m^6^A modification of *ATF4* mRNA

m^6^A is regulated by an RNA methyltransferase complex containing methyltransferase like 3 (METTL3), methyltransferase like 14 (METTL14), and WT1 associated protein (WTAP), as well as the m^6^A demethylases FTO and AlkB homolog 5, RNA demethylase (ALKBH5) 22.

**Figure 5:**
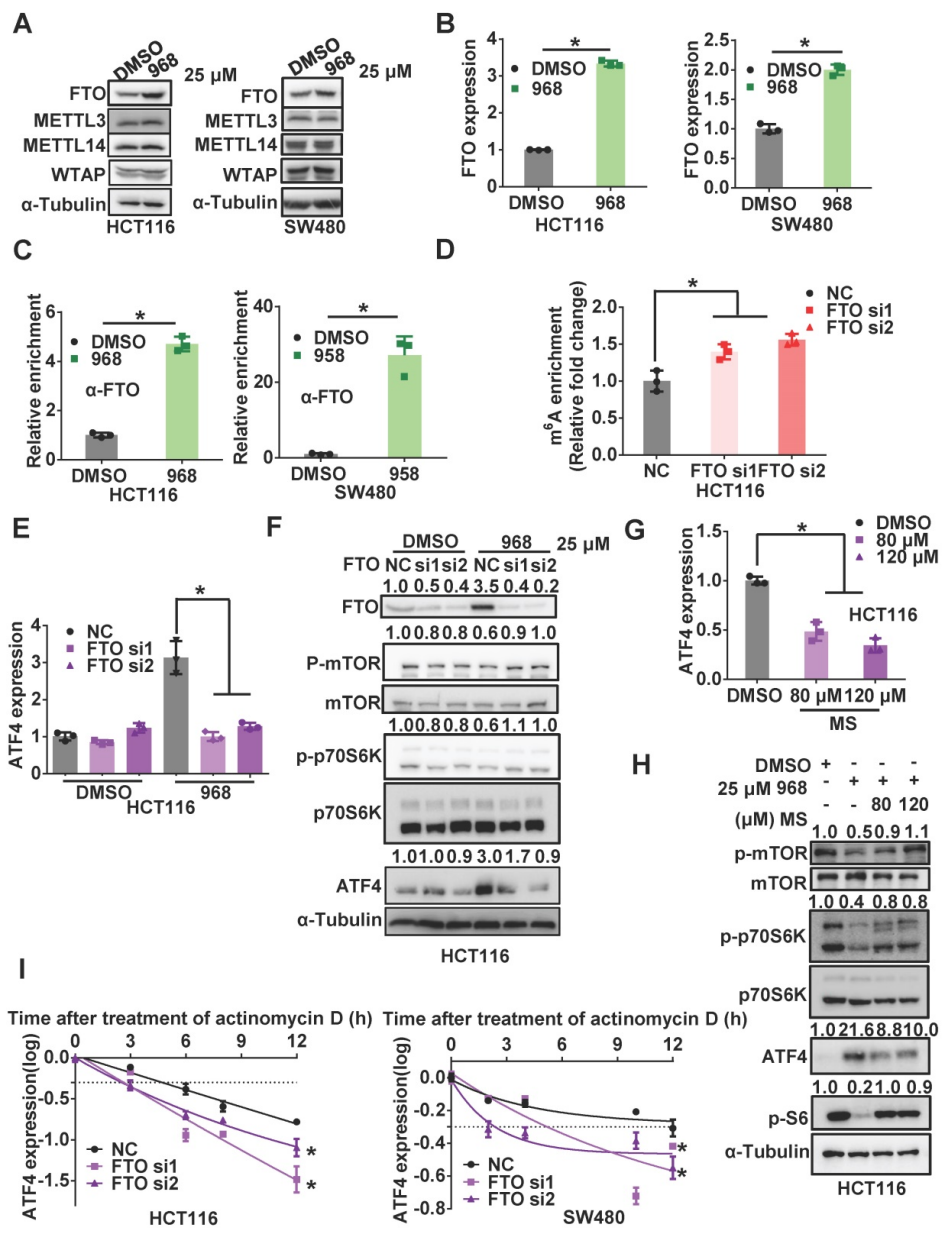
** Inhibition of glutaminolysis upregulates FTO to reduce m^6^A modification of *ATF4* mRNA.** (A) The effect of 968 treatment on protein expression of FTO, METTL3, METTL14, WTAP was detected by western blotting. (B) The effect of 968 on *FTO* mRNA expression was detected by qRT-PCR. (C) The effect of 968 on FTO enrichment at *ATF4* mRNA was assessed by RIP assay. (D) The effect of FTO knockdown on *ATF*4 mRNA m^6^A modification level was assessed by RIP assay. (E) The effect of FTO knockdown on* ATF4* mRNA expression during 968 treatment was detected by qRT-PCR. (F) The effect of FTO knockdown and on ATF4 protein expression and mTORC1 activity upon 968 treatment was detected by western blotting. p-mTOR, p-p70S6K, ATF4, p-S6 band density was quantified and expressed as fold change, compared with the control, by arbitrarily setting the control value as 1. (G) The effect of FTO inhibitor MS (80 μM and 120 μM) on* ATF4* mRNA expression during 968 treatment was detected by qRT-PCR. (H) The effect of FTO inhibitor MS (80 μM and 120 μM) on ATF4 protein expression and mTORC1 activity during 968 treatment was detected by western blotting. p-mTOR, p-p70S6K, ATF4, p-S6 band density was quantified and expressed as fold change, compared with the control, by arbitrarily setting the control value as 1. (I) The change of the half-life of* ATF4* mRNA after FTO knockdown was assessed by qRT-PCR. Data are shown as the means ± SD from three experiments. Statistical significance was assessed by ANCOVA analysis in (I), for other experiments, statistical significance was assessed by Student's* t*-tests, *P < 0.05.

Among these regulators, we noticed the protein level of FTO was increased most significantly in both cell lines after compound 968 treatment, while ALKBH5 was upregulated only in HCT116 cells (Figure 5A and Figure S5A). Moreover, the level of *FTO* mRNA was also elevated after glutaminolysis inhibition (Figure 5B). Hence, we suspected FTO to be the main regulator of *ATF4* mRNA m^6^A modification in response to glutaminolysis inhibition. Indeed, RIP assays demonstrated an increased interaction between FTO and *ATF4* mRNA after glutaminolysis inhibition (Figure 5C). Knocking down FTO rescued the glutaminolysis inhibition-induced decline of *ATF4* mRNA m^6^A levels (Figure 5D), highlighting the relevance of FTO upregulation to the reduced *ATF4* mRNA m^6^A modification in response to glutaminolysis inhibition. Accordingly, FTO knockdown abolished compound 968-induced ATF4 upregulation at both protein and mRNA levels (Figure 5E-F and Figure S5D-E). The FTO inhibitor meclofenamate sodium (MS) 25 also efficiently reduced ATF4 expression and rescued the mTOR downregulation induced by compound 968. (Figure 5G-H and Figure S5F). In addition, the half-life of* ATF4* mRNA was shortened upon FTO knockdown (Figure 5I). As an m^6^A writer, METTL3 acts in an opposite role to FTO. Therefore, we knocked down METTL3 to test whether the half-life of *ATF4* mRNA was affected. However, knocking down METTL3 demonstrated a subtle impact on *ATF4* expression mRNA stability (Figure S5B-C). Since the expression of METTTL3 is not significantly influenced by glutaminolysis inhibition, we suspected that FTO, rather than METTL3, plays the main role in *ATF4* mRNA regulation. Collectively, these results suggest that FTO is upregulated to stabilize *ATF4* mRNA by reducing *ATF4* mRNA m^6^A modification during glutaminolysis inhibition.

### ATF4 induces autophagy during glutaminolysis inhibition

**Figure 6:**
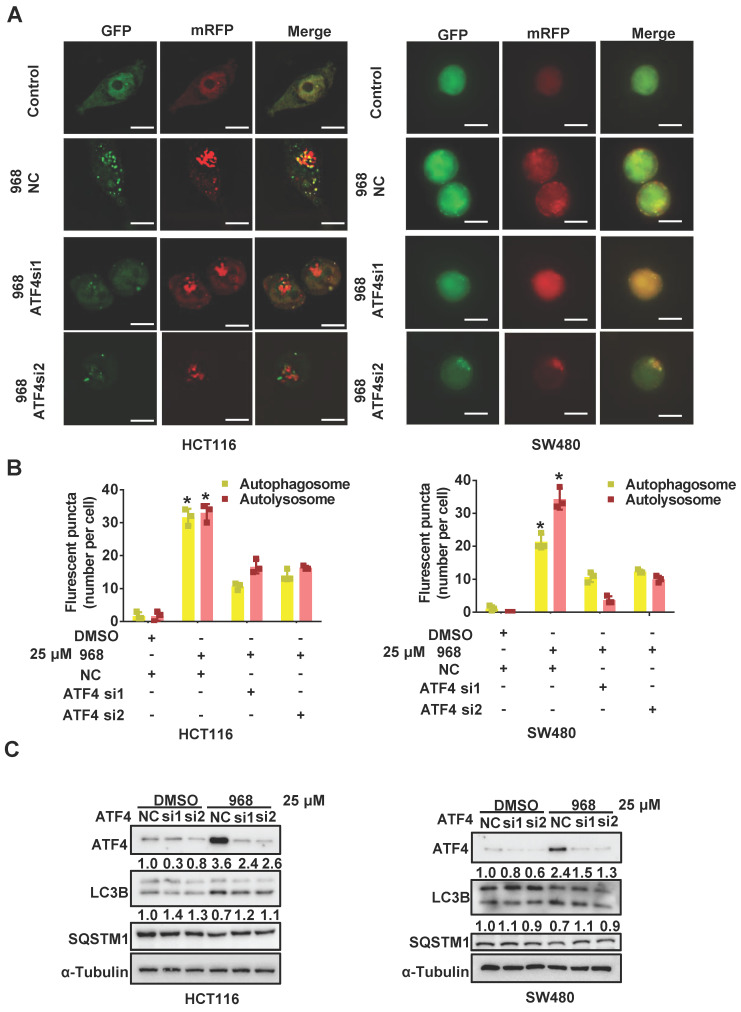
** ATF4 induces autophagy during glutaminolysis inhibition.** (A) HCT116 and SW480 cells were transfected with mRFP-GFP-LC3 plasmid and ATF4 siRNA. Then cells were treated with DMSO or Compound 968 respectively before subjected to confocal microscopy. (B) The average numbers of yellow (autophagosome) and red (autolysosome) LC3 dots per cell were counted with ImageJ. (C) The effect of ATF4 knockdown on expression of LC3 and SQSTM1 during 968 treatment was detected by western blotting. LC3-II/LC3-I ratio and SQSTM1 were quantified and expressed as fold change, compared with the control, by arbitrarily setting the control value as 1. Scale bars are 10 μm in (A). Data are shown as the means ± SD (B) from three experiments. For all experiments, statistical significance was assessed by Student's* t*-tests, *P < 0.05.

Since mTOR is a major negative regulator of autophagy, the activated ATF4 pathway may activate autophagy to promote cellular survival by suppressing mTOR activity. Therefore, we performed an autophagy assay using a tandem mRFP-GFP-tagged LC3 (tfLC3) reporter to detect the impact of ATF4 on autophagy during glutaminolysis inhibition. Since GFP is sensitive to acidic enzymes and mRFP is stable in acid environments like lysosomes, yellow puncta (GFP+/mRFP+) indicate autophagosome formation, while red puncta (GFP-/mRFP+) alone indicate for autolysosomes 26. Compared to the control group, compound 968-treated cells showed increased accumulation of yellow and red autophagic LC3 puncta. Therefore, glutaminolysis inhibition was sufficient to induce autophagy in HCT116 and SW480 cells. ATF4 knockdown reduced the formation of autolysosomes and autophagosomes, suggesting autophagy suppression (Figure 6A-B). Consistently, we detected the classic autophagy markers LC3B and SQSTM1 after ATF4 knockdown. ATF4 knockdown increased SQSTM1 accumulation and reduced LC3 conversion (Figure 6C). Taken together, these results indicate that ATF4 knockdown likely suppresses autophagy in its early stages. Hence, our results demonstrate that autophagy induced by glutaminolysis inhibition depends on the activated ATF4 axis.

### Targeting ATF4-dependent pro-survival autophagy to synergize glutaminolysis inhibition

**Figure 7:**
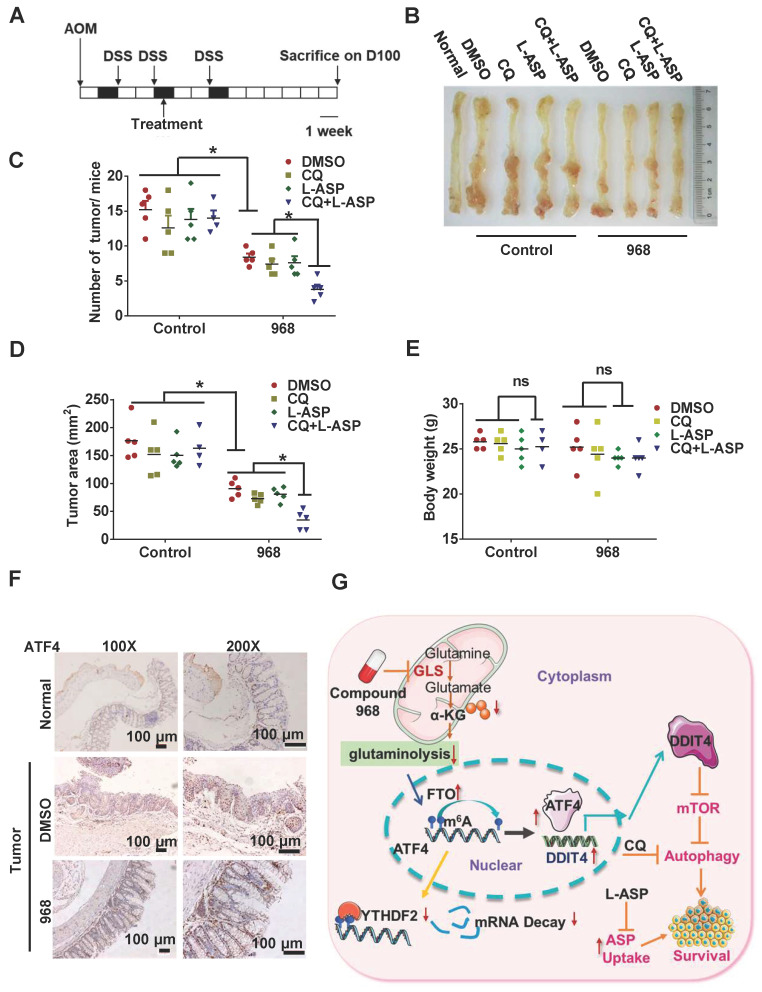
** Targeting ATF4-dependent pro-survival autophagy to synergize glutaminolysis inhibition.** (A-B) Timeline showing that mice were treated with AOM and with 2% DSS as indicated. From day 30 on, the mice began to be treated with respective drugs until sacrifice. On day 100, mice were euthanized and the tissue was collected as shown in (B). (C) Numbers of tumors per mouse were counted (n = at least 4, *P < 0.05). (D) Tumor size in mm^3^/tumor were measured (n = at least 4, *P < 0.05). (E) Body weights of mice were recorded (n = at least 4, *P < 0.05). (F) IHC analysis of ATF4 expression in normal tissue, DMSO and 968 treated tumors tissue. (G) Working model: during glutaminolysis inhibition, FTO enhances ATF4 expression by reducing YTHDF2-mediated mRNA decay. ATF4 transcriptionally upregulates DDIT4 to inhibit mTORC1 activity and promote pro-survival autophagy. Finally, targeting the ATF4 pathway by autophagy inhibition combined with asparagine inhibitor is synthetic lethality with glutaminolysis inhibitor in CRC. Data are shown as the means ± SD (C, D, E). For all experiments, statistical significance was assessed by Student's* t*-tests, *P < 0.05. Scale bars are 100 μm in (F).

Given the importance of glutaminolysis to tumor cell metabolism, targeting glutaminolysis has been proposed as a new intervention strategy for cancer therapy. During nutrient stress, autophagy can be activated to promote cell survival 27. In addition, cells under glutamine starvation can rely on exogenous asparagine as an alternative metabolic adaptation 28. Therefore, targeting asparagine usage is a necessary procedure to optimize the therapeutic efficacy of glutaminolysis inhibition 10. To explore the efficacy of this combinatorial therapy in vivo, we treated AOM/DSS-induced CRC model mice with an autophagy inhibitor (CQ), an asparagine inhibitor (L-ASP), and compound 968 (Figure 7A-B). While compound 968 alone had a moderate inhibitory effect on tumorigenesis, the addition of CQ and L-ASP significantly increased the efficacy of tumor inhibition (Figure 7C-D). Importantly, the combination therapy did not show a significant impact on mouse body weight (Figure 7E) or mobility (data not shown). Consistent with the in vitro results, ATF4 expression was upregulated in tumor tissues from mice treated with compound 968 (Figure 7F). Overall, inhibition of ATF4 driving pro-survival autophagy and glutaminolysis stress-adapted asparagine metabolism could be a promising strategy for synthetic lethality with glutaminolysis inhibitors in CRC (Figure 7G).

## Discussion

Tumor cells heavily rely on glutamine to fulfill their metabolic needs during continuous proliferation. The rate-limiting enzyme GLS, which turns glutamine into glutamate, is highly expressed in CRC cells 9. Thus, targeting glutaminolysis has become an important topic of anticancer research in recent years. So far, several compounds selectively targeting glutaminolysis, including CB-839 29, BPTES 30, and compound 968 31, have been explored for their potential in cancer treatment 10, 30, 32. Previously, we reported the therapeutic efficiency of compound 968 in CRC cells in vitro 10. Although compound 968 inhibited cell growth, it could inactivate mTOR signaling to induce pro-survival autophagy. The simultaneous inhibition of pro-survival autophagy could significantly improve the antitumor effect of glutaminolysis inhibition. In this study, we further revealed that glutaminolysis inhibition activates ATF4-dependent DDIT4 transcription to inactivate mTOR signaling.

mTOR signaling is a master regulator of cell growth and plays a central role in cell metabolism. Recent studies have identified amino acids as some of the most potent activators of mTOR signaling 33. In particular, glutamine was reported to promote mTOR translocation and activation at the lysosome membrane 15. In this study, we found that glutaminolysis inhibition by compound 968 resulted in inactivation of mTOR signaling in a time-dependent manner, although the intracellular glutamine content remained stable (Figure S1D). Therefore, other mechanisms apart from reducing the amino acid supply are responsible for the inactivation of mTOR regulation during glutaminolysis inhibition. It was previously reported that glutaminolysis inhibition could downregulate mTORC1 through egl-9 family hypoxia-inducible factors (EGLNs) inactivation and reactive oxygen species (ROS) production 34. In this study, through pathway analysis, we focused on the stress-induced transcription factor *ATF4*, which is a stress-induced gene triggered by hypoxia 35, endoplasmic reticulum stress and amino acid deprivation 36. ATF4 has been reported to be activated by amino acid deprivation 37. Interestingly, it is activated under such circumstances to promote mTORC1 activation. ATF4 can activate mTOR signaling through various mechanisms in different cell types. For example, it activates the mTORC1 pathway by upregulating the amino acid transporter solute carrier family 7 member 5 (SLC7A5) 38. In K-Ras-driven cancer cells, ATF4 activates the mTORC1 pathway by promoting the transcription of asparagine synthase (ASNS) for apoptosis suppression during glutamine deprivation 39. In contrast, during leucine deprivation, ATF4 upregulates the mTOR suppressors DDIT4 and sestrin 2 to inhibit mTOR activation 40. Taken together, these studies suggest that ATF4 plays a dual role in mTOR regulation in a context-dependent manner. Hence, understanding the determinants of ATF4 function during glutaminolysis inhibition may shed insights into mTOR regulation during metabolic stress. In this study, we found that ATF4 and its target DDIT4 were upregulated by glutaminolysis inhibition. DDIT4 has been previously found to inhibit mTOR by releasing the mTOR suppressor TSC2 from the 14-3-3 protein complex. DDIT4 transcription was activated by ATF4 upon glutaminolysis inhibition. Knocking down either ATF4 or DDIT4 restored mTOR activity during compound 968 treatment. Thus, our results suggest that glutaminolysis inhibition downregulates mTOR signaling through the ATF4-DDIT4 axis in CRC cells.

As a common mRNA modification, m^6^A modification can be recognized by distinct readers in an unknown manner to decide the fate of mRNAs. For example, insulin-like growth factor-binding protein 3 (IGFBP3) recognizes m^6^A modification of the long non-coding RNA* AS-ARHGAP5* to promote its degradation by RNautophagy 41, while YTHDF1 interacts with heat shock factor 1 (HSF1) mRNA with m^6^A modification to facilitate its translation 42. YTHDF2 promotes RNA decay through localizing target RNAs from the translating pool to processing bodies 43 and directing recruitment of the carbon catabolite repression 4-negative on TATA-less (CCR4-NOT) complex to target RNAs 44. Here we found that m^6^A plays an important role in ATF4 upregulation during glutaminolysis inhibition. In our study, we noticed elevation of *ATF4* mRNA levels and an extended *ATF4* mRNA half-life during glutaminolysis inhibition. Therefore, we presented stress-induced ATF4 upregulation as an alternative mechanism to protein translation. In contrast to previous reports that *ATF4* mRNA contains m^6^A modifications in its 5'-UTR to promote translation 45, we found that m^6^A modifications of *ATF4* mRNA also occur in its coding sequence which likely contribute to the regulation of RNA stability. In response to glutaminolysis inhibition, m^6^A modification of *ATF4* mRNA was decreased to abrogate its decay. During glutaminolysis inhibition, interaction of the RNA decay-related reader YTHDF2 with *ATF4* mRNA was decreased, and knocking down YTHDF2 prolonged the half-life of* ATF4* mRNA. Overall, our results suggest that m^6^A enrichment leads to *ATF4* RNA decay in a YTHDF2-dependent mechanism in CRC, which plays an important role in stress response in addition to the protein translation of ATF4 observed in other contexts. However, the m^6^A writer for *ATF4* mRNA m^6^A modification remains unknown. Knocking down METTL3 only moderately extended the half-life of *ATF4* mRNA, although this could be due to insufficient knockdown. Alternatively, other m^6^A writers, such as METTL16, could be responsible for *ATF4* mRNA m^6^A modification.

It remains unclear how RNA m^6^A levels change in response to metabolic stresses. It has been reported that the m^6^A eraser FTO is upregulated by metabolic stress (serum starvation and HBSS treatment) and plays a pro-tumorigenic role in melanoma cells 46. In addition, FTO destabilizes mRNA through m^6^A regulation 47. Our results demonstrate that FTO is upregulated by glutaminolysis inhibition in CRC cells to stabilize* ATF4* mRNA. By reducing the level of m^6^A, FTO might hinder YTHDF2 binding to *ATF4* mRNA, thus acting as a metabolic sensor. Indeed, FTO was found to sense essential amino acids and activate mTOR signaling in several cell types 48. However, we noticed that, during glutaminolysis inhibition, FTO suppressed mTORC1 activation by increasing ATF4 expression. Certainly, upregulation of FTO expression would presumably influence the m^6^A landscape in addition to *ATF4* mRNA. In doing so, FTO might play a central role in coupling metabolic signals to post-transcriptional gene expression on a genome-wide scale. Possibly, FTO plays different roles during energy stress depending on whether the cell is undergoing continuous or acute stress by inducing corresponding groups of genes. Additionally, glutaminolysis inhibition promotes ROS production by reducing glutathione production 49. We noticed that treatment of CRC cells with compound 968 also downregulated GSH (Figure S1E). Through oxidation, ROS are able to regulate various signals which might participate in the regulation of FTO activity and expression. FTO could in turn promote ROS production 50, which might lead to a positive regulation loop between ROS and FTO. Nevertheless, the regulation of FTO expression and function under such circumstances warrants further investigation to finally understand the regulation of m^6^A modification in response to stress.

Due to their massive consumption of circulating glutamine, tumor cells may frequently encounter glutamine deficiencies in the tumor environment 48, 51. As an adaption to glutamine deficiency, tumor cells activate autophagy to enable cell survival upon glutaminolysis inhibition 52, 53. Therefore, autophagy should be inactivated to improve the clinical efficacy of molecular therapies targeting glutaminolysis. Earlier studies showed that cells lacking ATF4 are more vulnerable to amino acid starvation 54. In addition, ATF4 induces pro-survival autophagy by promoting transcription of *SQSTM1/p62* 54 and other core autophagy genes such as microtubule-associated protein 1 light chain 3 beta (*MAP1LC3B*) 55 and beclin 1 (*BECN1*) 56. In this study, we found that ATF4 activates the transcription of DDIT4 to inactivate mTOR signaling and induce autophagy. Depleting ATF4 expression inhibited the autophagy activation induced by glutaminolysis inhibition. Accordingly, DDIT4 knockdown increased the apoptosis activated by glutaminolysis inhibition. Since a selective inhibitor of ATF4 has yet to be developed, autophagy inhibition could be a valid approach for cancer treatment. For example, the classic autophagy inhibitor CQ, which has been clinically used for the treatment of malaria, was shown to be effective in sensitizing cells to glutaminolysis inhibition 10 and hypoxia 56. More importantly, we demonstrated that autophagy inhibition synergizes with the GLS inhibitor compound 968 in vivo. Nevertheless, similar to the dual role of ATF4, the effects of autophagy on cell viability are also complicated. Autophagy has been shown to play a pro-survival role by promoting catabolism and maintaining cellular balance. For cells that rely heavily on glutamine 57, and cells with high basal autophagy levels 58, studies have shown that autophagy is activated even under normal conditions for maintenance of intracellular glutamine levels 57. Under such circumstances, co-inhibition of autophagy and glutaminolysis was shown to result in complete blockage of glutamine supply and cell apoptosis, which is similar to our results. However, if cell stress is not restored, autophagy plays a pro-necroptotic role. In glutaminolysis-thriving ovarian cancer cells, autophagy induction and GLS1 inhibition lead to increased cell death 59. Therefore, whether dual autophagy/GLS1 inhibition is beneficial or not is tightly related to tumor specificity, glutaminolysis activity, and stress tolerance. Given these contradictions, more studies are needed to investigate the regulation of this “switch” between the pro- and anti-survival roles of autophagy.

In summary, glutaminolysis inhibition upregulates the m^6^A eraser FTO to reduce m^6^A modification of *ATF4* mRNA and extend its half-life by preventing its degradation in CRC cells. As a result, upregulated ATF4 expression activates transcription of DDIT4 to inactivate mTOR signaling and induce pro-survival autophagy, which should be simultaneously inhibited to synergize with glutaminolysis inhibition.

## Supplementary Material

Supplementary figures and tables.

## Abbreviations

CRC: colorectal cancer
ATF4: activating transcription factor 4
DDIT4: DNA damage inducible transcript 4
mTOR: mechanistic target of rapamycin
GSEA: gene set enrichment analysis
ChIP: chromatin immunoprecipitation
RIP: RNA immunoprecipitation
GLS1: glutaminase 1
m^6^A: N^6^-methyladenosine
FTO: FTO alpha-ketoglutarate dependent dioxygenase
ALKBH5: AlkB homolog 5, RNA demethylase
METTL3: methyltransferase like 3
METTL14: methyltransferase like 14
WTAP: WT1 associated protein
YTHDF2: YTH N^6^-methyladenosine RNA binding protein 2
IHC: immunohistochemical
DMSO: dimethyl sulfoxide
968: compound 968
CQ: chloroquine
L-ASP: L-asparaginase
MS: meclofenamate sodium
ROS: reactive oxygen species
GSH: glutathione
