# Supplementary material for: Targeting ATF4-dependent pro-survival autophagy to synergize glutaminolysis inhibition
Source: Theranostics. 2021 Jul 25;11(17):8464–79. doi: 10.7150/thno.60028 (PMC8343999; doi:10.7150/thno.60028)
Supplement: Supplementary file 1 — Supplementary figures and tables. [file thnov11p8464s1.pdf]

Supplemental Figures

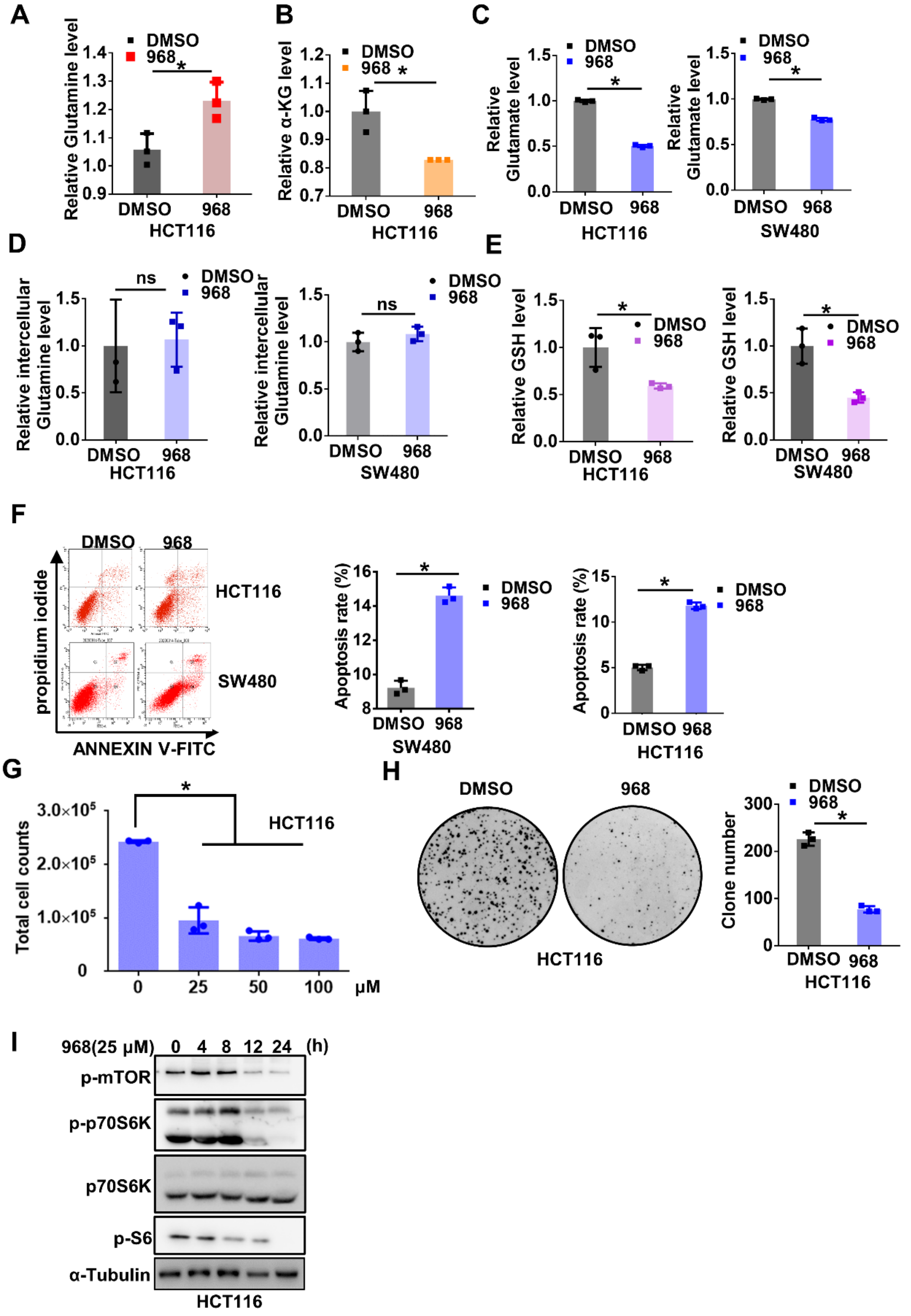

Figure S1 Inhibition of glutaminolysis inactivates mTOR

(A–E) The glutamine level in medium (A), Intercellular level of α-KG (B), glutamate (C), Intercellular level of glutamine (D), and Intercellular level of glutathione (GSH) (E) of cell treated by DMSO or 968 (25 μM) for 24 h were detected by metabolic analysis. (F) Apoptosis induced by 968 or DMSO was detected by Flow Cytometry. The result presents the proportion of apoptotic cells. (G) The effect of 968 on cell viability was determined by cell counting assay. Cells treated with DMSO or 968 (25 μM, 50 μM,100 μM) for 72 h were then counted. Data are shown as the means ± SD from three experiments. (H) The colony formation of cells treated with 968 at 25 μM. The result presents the number of colonies. (I) The effect of 968 on mTORC1 activity was detected by western blotting. For all experiments, statistical significance was assessed by Student's *t*-tests, $^{*}P < 0.05$.

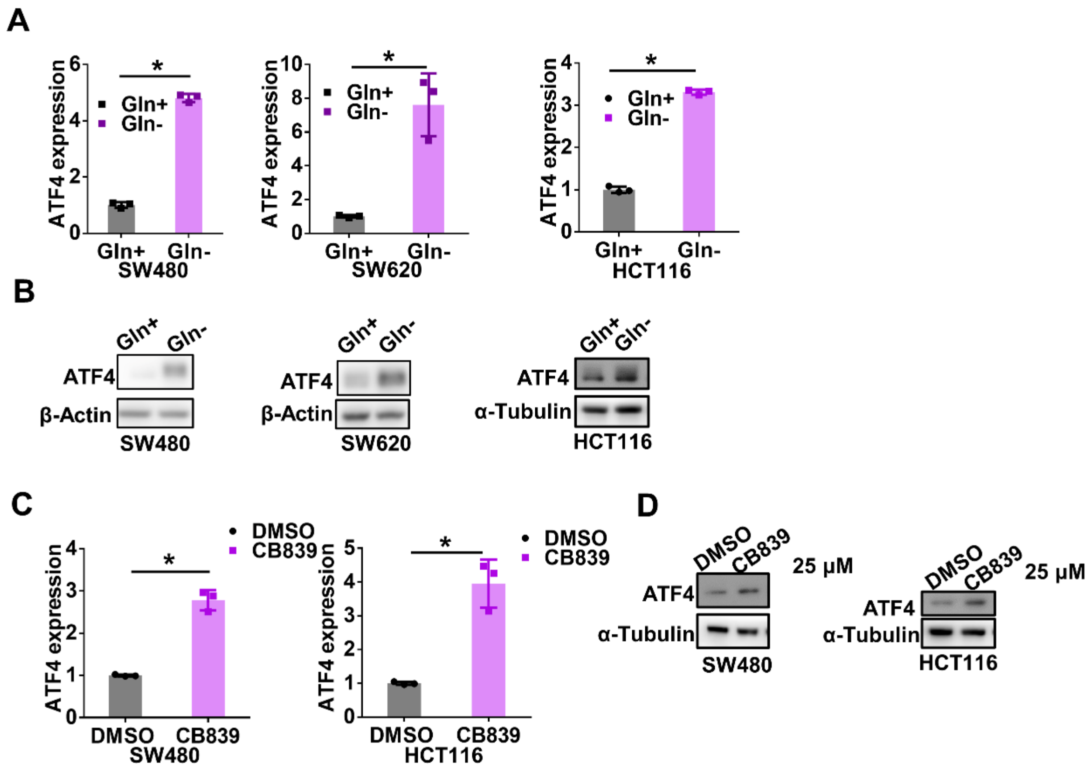

Figure S2 Inhibition of glutaminolysis upregulates ATF4 expression

(A–B) ATF4 expression of cells treated with glutamine starvation for 24h was detected by qRT-PCR (A) and western blotting (B). (C–D) ATF4 expression of cells treated with CB839 (25 μM) for 24 h was detected by qRT-PCR (C) and western blotting (D). Data are shown as the means ± SD from three experiments. For all experiments, statistical significance was assessed by Student's *t*-tests, *$P < 0.05$.

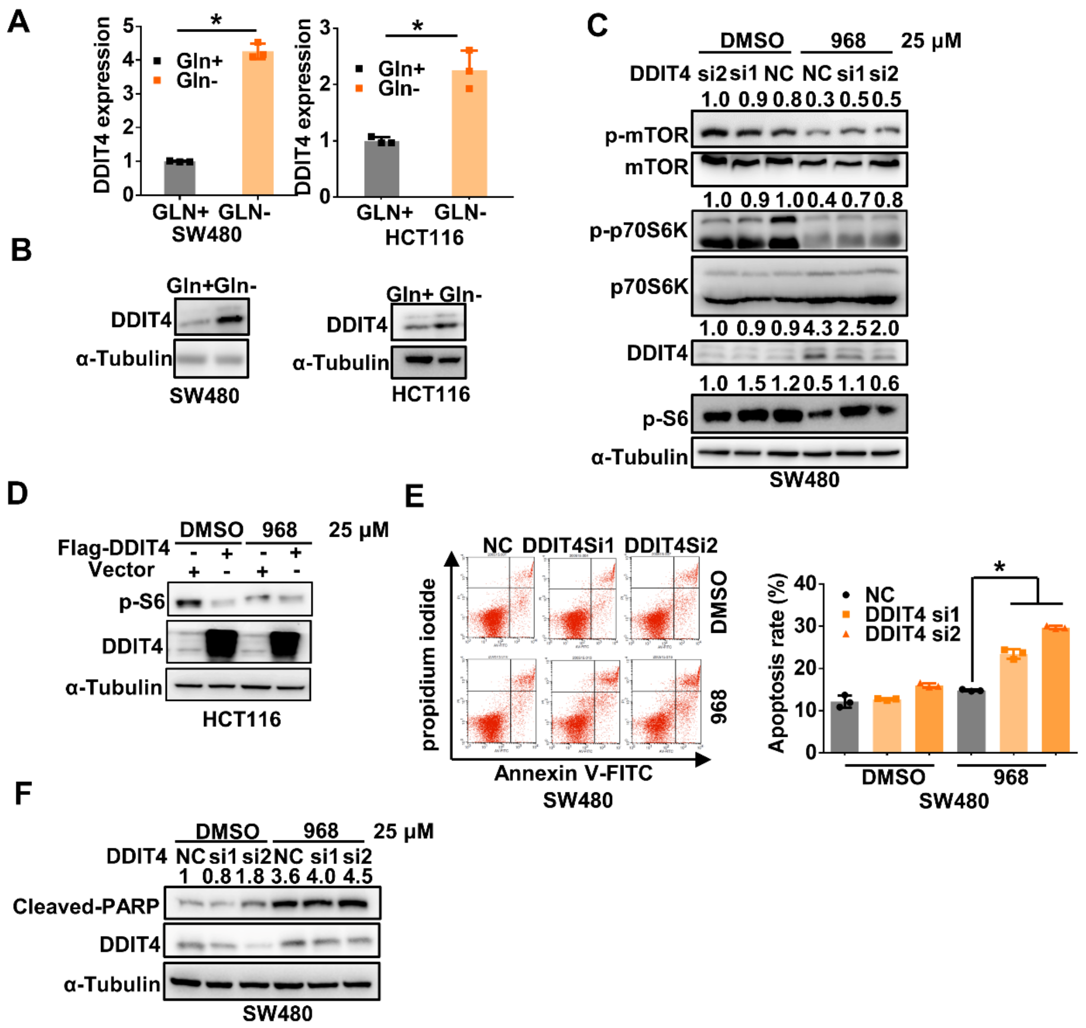

Figure S3 Inhibition of glutaminolysis upregulates DDIT4 to inactivate mTOR

(A–B) DDIT4 expression of cells treated by glutamine starvation for 24 h was detected by qRT-PCR and western blotting (B). (C) The effect of DDIT4 knockdown on mTORC1 activity with 968 treatment was detected by western blotting. p-mTOR, p-p70S6K, p-S6, DDIT4 band density was quantified and expressed as fold change, compared with the control, by arbitrarily setting the control value as 1. (D) The effect of DDIT4 overexpression on mTORC1 activity in HCT116 with 968 treatment was detected by western blotting. (E) The effect

of DDIT4 knockdown on 968-induced cell apoptosis in SW480 cells was measured by Flow Cytometry. The result presents the proportion of apoptotic cells. (F) The expression of cleaved PARP after DDIT4 knockdown and 968 treatment was detected by western blotting, cleaved PARP band density was quantified and expressed as fold change compared with the control, by arbitrarily setting the control value as 1. Data are shown as the means ± SD from three experiments. For all experiments, statistical significance was assessed by Student's *t*-tests, $*P < 0.05$.

A

ATF4 putative sites on DDIT4 promoter				
TF	Score	Start	End	Strand
ATF4	7.837	830	842	1
ATF4	13.119	995	1007	1
ATF4	13.207	1057	1069	1
ATF4	5.819	2166	2178	-1

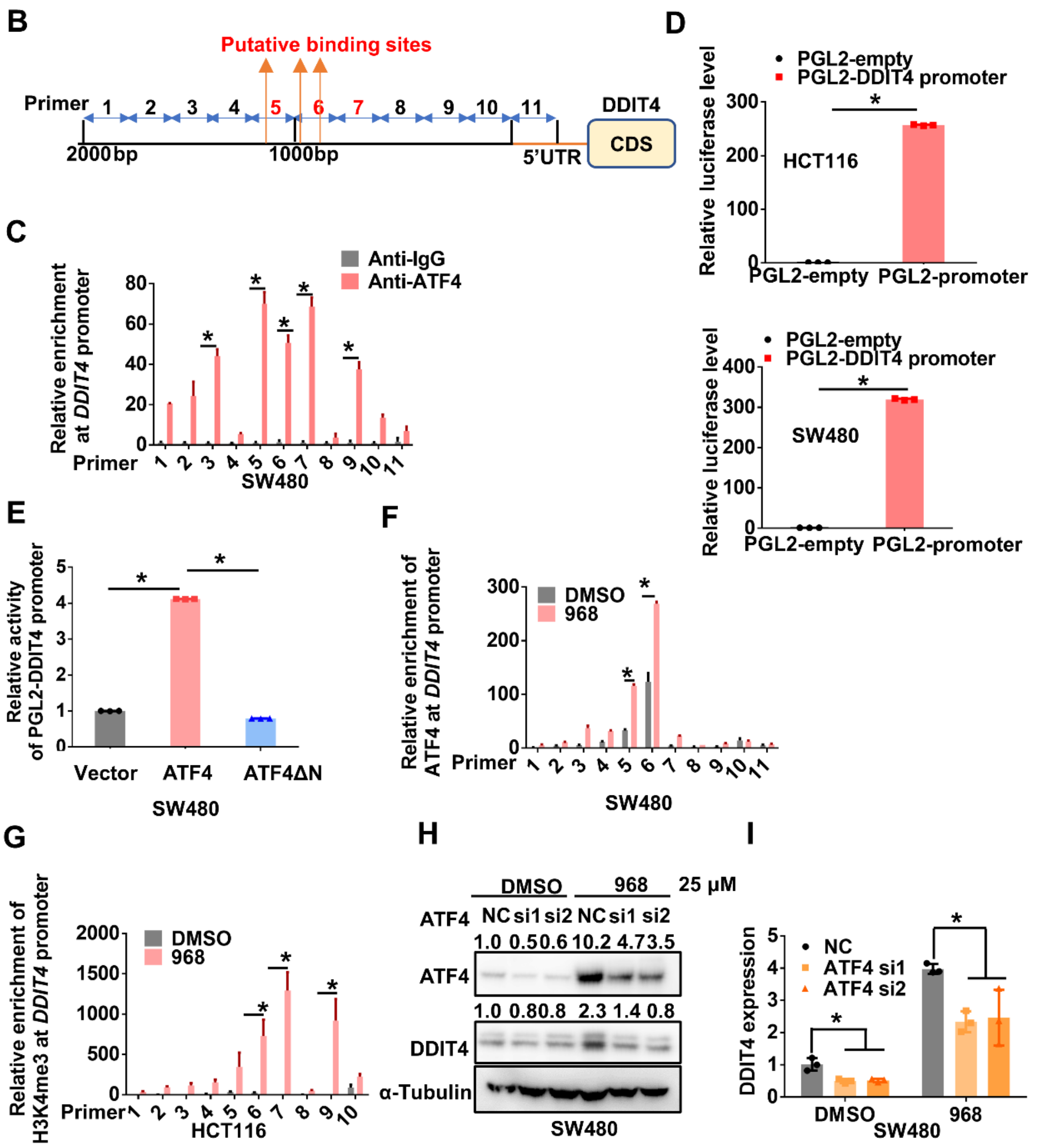

Figure S4 ATF4 transcriptionally upregulates DDIT4 upon inhibition of glutaminolysis

(A) Putative binding sites of ATF4 on the *DDIT4* promoter were generated through an online database called JASPAR. (B) Putative ATF4 binding sites and the *DDIT4* promoter primers design were shown as indicated. (C) ATF4 enrichment at the *DDIT4* promoter was determined by ChIP assay. (D) The Compared transcription ability of PGL2-DDIT4 promoter and PGL2-empty was measured by luciferase assay. (E) The transcription activation ability of ATF4 and ATF4ΔN on the *DDIT4* promoter was measured by luciferase assay. (F) The change of ATF4 enrichment at the *DDIT4* promoter after 968 treatment was measured by luciferase assay. (G) The change of H3K4me3 enrichment at the *DDIT4* promoter in HCT116 was determined with ChIP assay. (H) DDIT4 protein expression after ATF4 knockdown upon 968 treatment was detected by western blotting. ATF4, DDIT4 band density was quantified and expressed as fold change, compared with the control, by arbitrarily setting the control value as 1. (I) *DDIT4* mRNA expression after ATF4 knockdown during 968 treatment was detected by qRT-PCR. Data are shown as the means ± SD from three experiments. For all experiments, statistical significance was assessed by Student's *t*-tests, *$P < 0.05$.

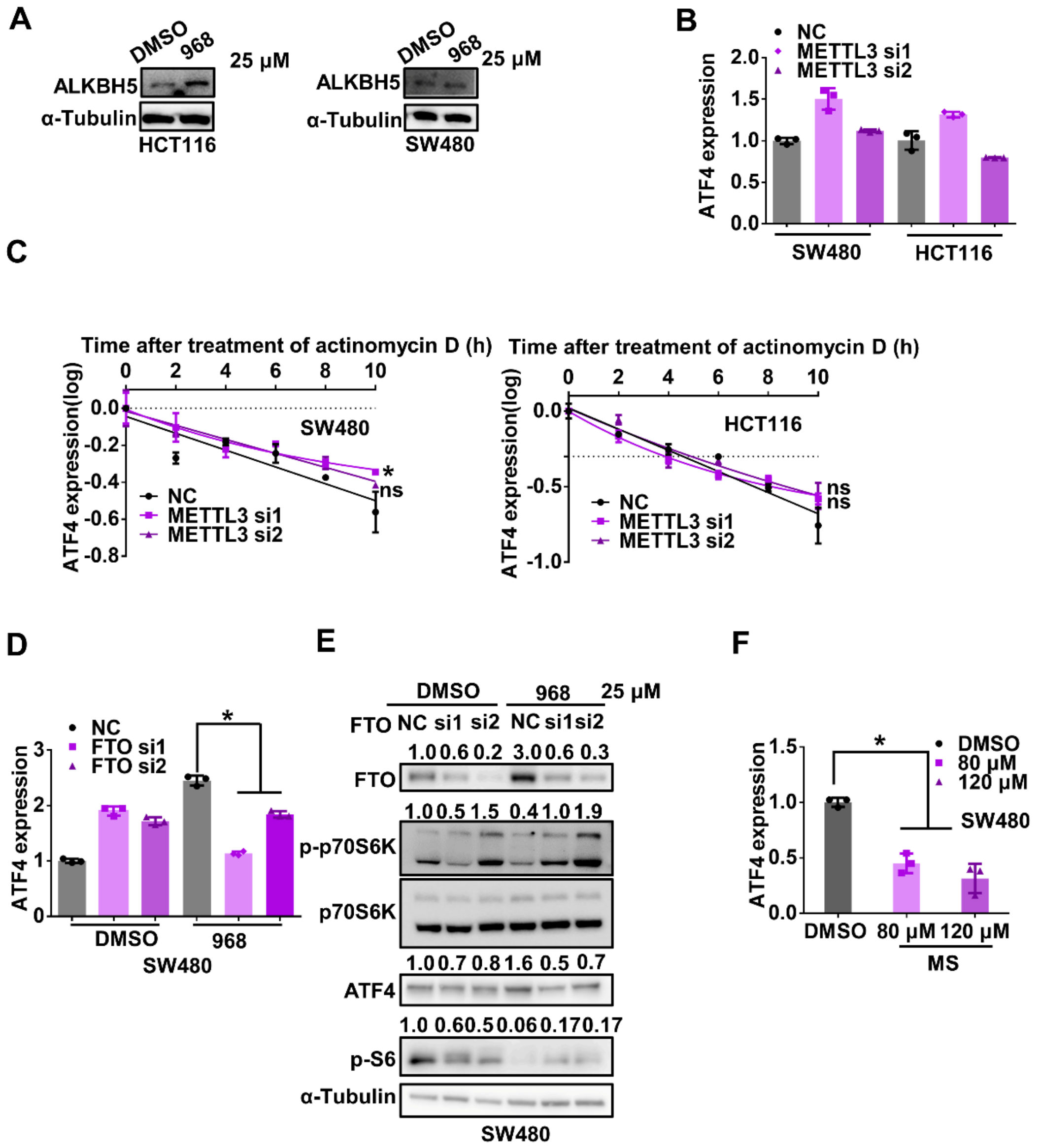

Figure S5 FTO stabilizes ATF4 mRNA by reducing its m^{6}A modification during glutaminolysis inhibition

(A) The effect of 968 treatment on ALKBH5 protein expression was detected by western blotting. (B) The effect of METTL3 knockdown on *ATF4* mRNA expression was detected by qRT-PCR. (C) The effect of METTL3 knockdown on *ATF4* mRNA stability was detected by qRT-PCR. (D) The effect of FTO knockdown on *ATF4* mRNA expression upon 968 treatment was detected with qRT-PCR. (E) The effect of FTO knockdown and on ATF4 protein expression

and mTORC1 activity upon 968 treatment was detected by western blotting. p-p70S6K, ATF4, p-S6 band density was quantified and expressed as fold change, compared with the control, by arbitrarily setting the control value as 1. (F) The effect of FTO inhibitor MS (80 μM and 120 μM) on *ATF4* mRNA expression upon 968 treatment was detected by qRT-PCR. Data are shown as the means ± SD from three experiments. For all experiments, statistical significance was assessed by Student's *t*-tests, $^{*}P < 0.05$, ns, not significant.

Table S1

Primers	
β-actin-F	CACCAACTGGGACGACAT
β-actin-R	ACAGCCTGGATAGCAACG
ATF4-F	CTTAAGCCATGGCGCTTCTC
ATF4-R	GAAGGCATCCTCCTTGCTGTT
DDIT4-F	GCTAGCTGCGGCTTCTACG
DDIT4-R	CAGGTAAGCCGTGTCTTCCTC
FTO-F	GATGGAGGGTGTGACAAATGC
FTO-R	CCTTTTCCCAGTATGGCCGA
DDIT4 pomoter-1 F	GTGGGCACCTGCAGTTC
DDIT4 pomoter-1 R	CCTAAGGAAACAAGAGTTACGATG
DDIT4 pomoter-2 F	TGTCCCGCCCAAATC
DDIT4 pomoter-2 R	TAATAAAAGACACTGGATCAAGGC
DDIT4 pomoter-3 F	CCCCATTGTTAAGCAATCTG
DDIT4 pomoter-3 R	CCACTTATGG AGACAAATTTATGG
DDIT4 pomoter-4 F	GTCAAATGAGAGTAGGGTGCAG
DDIT4 pomoter-4 R	CTAACCAGATAGGCGGGC
DDIT4 pomoter-5 F	GCCCTGGGCATCTGAT
DDIT4 pomoter-5 R	TGGCAGCAGTGCTCAGA
DDIT4 pomoter-6 F	GCCTGAATGATGAAACACGG
DDIT4 pomoter-6 R	TCGGACTCAC AGACCCATC
DDIT4 pomoter-7 F	GTGAATCCTCGCTTCCATC
DDIT4 pomoter-7 R	CCATCCTGGCTGTTACTCTACAG
DDIT4 pomoter-8 F	CCCGGAGCTTCCCAGTC
DDIT4 pomoter-8 R	GGAACAACTGGGCACACAC
DDIT4 pomoter-9 F	AACCATTTTCCTTGCCCGC

DDIT4 pomoter-9 R	CTGCCAAGGTCCCCGAG
DDIT4 pomoter-10 F	GGTTCGACTGCGAGCTTT
DDIT4 pomoter-10 R	CCTTCTCTGCGCCACGA
DDIT4 pomoter-11 F	GAGCGTGGACCTGGGAC
DDIT4 pomoter-11 R	GGGCGTTTGCTGATGAACTC
DDIT3-F	GGAAACAGAGTGGTCATTCCC
DDIT3-R	CTGCTTGAGCCGTTCATTCTC
ASNS-F	GGAGCCAGGTCGGTATAAGC
ASNS-R	CCGGTGAAATCCAAAGCAGC
NUPR1-F	CTCTCATCATGCCTATGCCTACT
NUPR1-R	CCTCCACCTCCTGTAACCAAG
PHGDH-F	CACGACAGGCTTGCTGAATGA
PHGDH-R	CTTCCGTAAACACGTCCAGTG
ShYTHDF2-1 F	CCGGGCTACTCTGAGGACGATATTCCTCGAGGAATATCGTC CTCAGAGTAGCTTTTTG
ShYTHDF2-1 R	AATTCAAAAAGCTACTCTGAGGACGATATTCCTCGAGGAATAT CGTCCTCAGAGTAGC
ShYTHDF2-2 F	CCGGCGGTCCATTAATAACTATAACCTCGAGGTTATAGTTATTA ATGGACCGTTTTTG
ShYTHDF2-2 R	AATTCAAAAACGGTCCATTAATAACTATAACCTCGAGGTTATAGT TATTAATGGACCG

Table S2

SiRNAs	
Control siRNA S	UUCUCCGAACGUGUCACGUTT
Control siRNA AS	ACGUGACACGUUCGGAGAATT
ATF4-1 S	CUGCUUACGUUGCCAUGAUTT
ATF4-1 AS	AUCAUGGCAACGUAAGCAGTT
ATF4-2 S	CCAAAUAGGAGCCUCCCAUTT
ATF4-2 AS	AUGGGAGGCUCCUAUUUGGTT
DDIT4-1 S	CCAGGUGGGCAAAGAACUATT
DDIT4-1AS	UAGUUCUUUGCCCACCUGGTT
DDIT4-2 S	CUGGCUUCCGAGUCAUCAATT
DDIT4-2 AS	UUGAUGACUCGGAAGCCAGTT
FTO-1 S	ACACUUGGCUCCCUUAUCUTT
FTO-1 AS	AGAUAAGGGAGCCAAGUGUTT
FTO-2 S	GUGGCAGUGUACAGUUAUATT
FTO-2 AS	UAUAACUGUACACUGCCACTT
YTHDF2-1 S	CCUACCAGAUGCAAUGUUUTT
YTHDF2-1 AS	AAACAUUGCAUCUGGUAGGTT
YTHDF2-2 S	GCUCCUGGCAUGAAUACUATT
YTHDF2-2 AS	UAGUAUUCAUGCCAGGAGCTT
METTL3-1 S	CCUGCAAGUAUGUUCACUATT
METTL3-1 AS	UAGUGAACAUACUUGCAGGTT
METTL3-2 S	GCUACCUGGACGUCAGUAUTT
METTL3-2 AS	AUACUGACGUCCAGGUAGCTT
